# PCSK9: A Potential Therapeutic Target for Sepsis

**DOI:** 10.1155/2020/2687692

**Published:** 2020-10-14

**Authors:** Yuan Yuan, Wei Wu, Shanshan Sun, Yi Zhang, Zhi Chen

**Affiliations:** ^1^Department of Neurology, The First Affiliated Hospital, College of Medicine, Zhejiang University, Hangzhou 310003, China; ^2^State Key Laboratory for Diagnosis and Treatment of Infectious Diseases, National Clinical Research Center for Infectious Diseases, Collaborative Innovation Center for Diagnosis and Treatment of Infectious Diseases, The First Affiliated Hospital, College of Medicine, Zhejiang University, Hangzhou 310003, China; ^3^Department of Laboratory Medicine, The First Affiliated Hospital, College of Medicine, Zhejiang University, Hangzhou 310003, China; ^4^Key Laboratory of Clinical In Vitro Diagnostic Techniques of Zhejiang Province, Hangzhou 310003, China

## Abstract

Sepsis is a life-threatening organ dysfunction syndrome caused by a dysregulated host response to infection. Proprotein convertase subtilisin/kexin type 9 (PCSK9) is often upregulated in the presence of sepsis and infectious diseases. In sepsis, PCSK9 degraded the low-density lipoprotein cholesterol (LDL) receptors (LDL-R) of the hepatocytes and the very low-density lipoprotein cholesterol receptors (VLDL-R) of the adipocytes, which then subsequently reduced pathogenic lipid uptake and clearance/sequestration. Moreover, it might improve cholesterol accumulation and augment toll-like receptor function in macrophages, which supported inflammatory responses. Accordingly, PCSK9 might show detrimental effects on immune host response and survival in sepsis. However, the exact roles of PCSK9 in the pathogenesis of sepsis are still not well defined. In this review, we summarized the literatures focusing on the roles of PCSK9 in sepsis. Our review provided an additional insight in the role of PCSK9 in sepsis, which might serve as a potential target for the treatment of sepsis.

## 1. Introduction

Sepsis is a life-threatening organ dysfunction syndrome caused by a dysregulated host response to infection [1]. Despite advances in the diagnosis and treatment, it is still a challenge with high mortality in an intensive care unit. To date, extensive studies have confirmed that cholesterol and pathogen lipids play crucial roles in the pathogenesis of sepsis. Pathogen lipid moieties such as lipopolysaccharide (LPS), lipoteichoic acid, and phospholipomannan are major ligands for innate immune receptors such as Toll-like receptors (TLRs). Cholesterol is a main component of lipid rafts that also involves in amplification of TLR signaling [2–4]. Increased TLR activity leads to augmented production of cytokines and chemotactic factors and amplification of the inflammatory process. However, the exact mechanisms on the TLR activation during sepsis are still not clear.

Nowadays, extensive efforts have been made to investigate the roles of proprotein convertase subtilisin/kexin type 9 (PCSK9) serving as the 9th member of the serine proteinase family [5–7]. It was initially believed to show a similar function with the other proprotein convertase members; however, it was then reported to present a specialized function to target the low-density lipoprotein cholesterol (LDL)/very low-density lipoprotein cholesterol (VLDL) receptors and involved in the degradation accordingly. It has been well acknowledged that hepatic uptake of cholesterol and pathogenic lipids is mediated by LDL receptors (LDL-R) and VLDL receptors (VLDL-R). Then, cholesterol and pathogenic lipids are excreted from hepatocytes into bile [5]. On this basis, PCSK9 is involved in the modulation of cholesterol and pathogenic lipid levels in sepsis. Ever since its discovery, many studies have been conducted to identify the potential mechanisms of how PCSK9 acts on the LDL-R and the onset of certain diseases [8, 9]. To our best knowledge, many studies verified the roles of PCSK9 in the pathogenesis of hypercholesterolemia and atherosclerosis and several PCSK9-based agents have been available, which then increased the level of LDL-R on the hepatocyte surface. Unfortunately, little is known about the roles of PCSK9 in inflammation and sepsis. In this review, we summarized the literatures on the roles of PCSK9 in these aspects.

## 2. Biology of PCSK9

PCSK9 is the 9th member of the proprotein convertase (PC) that involves in the protein activation, deactivation, and cellular localization [5–7]. The PC family has been reported to implicate with several proteins including proenzymes, prohormones, growth factors, and cytokines, as well as the adhesion molecules and the receptors. PCSK9 was initially identified in 2003 as a cause of autosomal familial hypercholesterolemia (FH) [7]. In the presence of apoptosis of cerebral neurons, the PCSK9 cDNA was upregulated to some extent. Thus, it was initially designated as the neural apoptosis-regulated convertase 1 (NARC-1) upon identification. To date, multiple transcriptional factors have been reported to regulate the expression of the *PCSK9* gene. For example, SREBP2 and HNF1*α* are involved in the positive regulation of the *PCSK9* gene [10], while the fibroblast growth factor 21 (FGF21) negatively controls its transcriptional expression [11].

The human *PCSK 9* gene is localized on the 1p32.3 adjacent to the genetic locus of the FH. It consists of 12 exons with a full length of 22 kb, encoding 692 amino acids. PCSK9 is generated from the soluble pro-pcsk9 with a molecular weight of 74 kDa through the autocatalysis in the endoplasmic reticulum, together with the release of the propeptide with a molecular weight of 14 kDa from the N-terminus, and the subsequent generation of the mature protein (60 kDa). Such self-slicing is crucial for the biological responses of PCSK9 and the maturity [5, 7]. To our best knowledge, it is extensively expressed in the liver, small intestine, and kidneys. The liver is the major target at which PCSK9 protein functions and also the major source of the circulating PCSK9.

Recent studies indicate a potential link between the PCSK9 protein and the cholesterol metabolism. The LDL-R on the surface of hepatocytes could bind with the LDL particles, forming the LDL-R-LDL complex. Therefore, the LDL-R on the cellular surface is crucial for the metabolism of LDL particles. Upon the entry of LDL-R-LDL complex into the cells, the LDL particles were separated with the LDL receptor. Then, the LDL particles were degraded into the cholesterol, which was stored in the hepatocytes. Afterwards, the LDL receptor would circulate into the cellular surface and involve in the uptake of LDL particles. Upon the maturation of the PCSK9 protein through autocatalysis in the endoplasmic reticulum (ER), it would be released into the peripheral circulation. Then, it forms the LDL-R complex upon binding with the EGF-A domain and reenters into the hepatocytes through the endocytosis. In the intracellular pathway, PCSK9 could bind the endocytosed LDL-R and direct the PCSK9-LDL-R-LDL complex to lysosomes for degradation [12, 13], and then, the degradation substances were discharged through the bile excretion which reduced the amount of LDL-R. Therefore, the PCSK9 protein plays an important role in the regulation of LDL and cholesterol metabolism. For instance, the gain-of-function (GOF) variation of PCSK9 was associated with the FH pathogenesis [11]. In addition, the patients with GOF mutation of the *PCSK9* gene showed higher risk of the coronary artery disease (CAD) in the FH patients compared to those with LDL-R mutation or apolipoprotein B (Apo B) mutation [14]. Among the population with loss-of-function (LOF) variation of *PCSK9*, there was a remarkable decline of serum LDL cholesterol (LDL-C) level as well as the prevalence of atherosclerosis [15].

The decline of intracellular free cholesterol would upregulate the sterol regulatory element-binding protein 2 (SREBP-2), which provides feedback to promote the expression of PCSK9, LDL-R, and HMG-CoA reductase protein [10, 16]. Statins could enhance the expression of LDL-R and inhibit the HMG-CoA reductase, which could promote the clearance of LDL in the liver [17, 18]. Interestingly, statins could induce the expression of PCSK9, which downregulate the expression of LDL-R. Therefore, there is not a linear negative correlation between the statin concentration and the LDL level. This could explain the similar results of high-dose (40 mg per day) and low-dose statins (10 mg per day) in decreasing the LDL-C (42% vs. 30%) [19].

## 3. PCSK9 as a Biomarker in the Prognosis of Sepsis

Sepsis refers to a critical illness induced by excessive responses to the infection by the host featured by multiple organ dysfunction syndromes in the distal infection sites [1]. Pathogen lipids released into the blood could trigger the systemic inflammatory response syndrome (SIRS). Nowadays, studies on the roles of PCSK9 in the infection mediated by pathogenic bacteria are mainly focused on sepsis.

In clinical studies, PCSK9 was considered to be crucial for the pathogenesis of sepsis, while inhibiting the PCSK9 activity could effectively improve the prognosis of sepsis [20–22]. Thus, PCSK9 may serve as a biomarker in the prognosis of sepsis. For instance, the serum PCSK9 level in sepsis patients showed significant elevation, which may be closely related to the subsequent multiple organ failure [20]. In another clinical trial, patients with septic shock who have at least one PCSK9 loss-of-function (LOF) allele showed increased survival over a 28-day period compared to patients without a LOF or a gain-of-function (GOF) allele (71.7% vs. 61.0%) [21]. Similarly, PCSK9 LOF variants were associated with an increased clearance of pathogen lipids, a decreased 1-year mortality, and recurrent infection in septic survivors [22]. Recently, the subanalysis of the Albumin Italian Outcome Sepsis (ALBIOS) study showed that patients with septic shock presenting with lower plasma PCSK9 levels experienced a higher 28- and 90-day mortality rate [23]. On the other hand, it is known that increased arterial stiffness is associated with clinical outcomes in patients with early sepsis. In the Brisighella Heart Study cohort, Ruscica et al. found that the circulating PCSK9 level was significantly related to arterial stiffness pleasured with pulse wave velocity, independent of sex and menopausal status in women [24].

In septic mice and wild-type, *PCSK9* knockout (KO), and transgenic (Tg) mice overexpressing PCSK9 subjected to sham surgery or cecal ligation and puncture (CLP), overexpression of PCSK9 in mice exacerbated liver and kidney pathology, as well as plasma IL-6, alanine aminotransferase (ALT), and thrombin-antithrombin (TAT) concentrations during sepsis, whereas the *PCSK9* KO mice exhibited reduced bacterial loads, lung and liver pathology, myeloperoxidase activity, and plasma level of IL-10 and cfDNA during CLP-induced sepsis [25]. Together with the sepsis induced by the Gram-negative bacteria, there were similar findings in the Gram-positive bacteria, in which the 28-day survival in the septic patients with *PCSK9 LOF* gene mutation was significantly higher than those without. Moreover, *PCSK9* KO mice showed improved serum lipoteichoic acid clearance compared to that of the wild type. Exactly, HepG2 hepatocytes pretreated with wild-type PCSK9 showed obvious decline in the lipoteichoic acid uptake than those with LOF variants of recombinant PCSK9 [26]. Nevertheless, there are still controversies on the roles of PCSK9 in the pathogenesis of sepsis. A recent study on a cohort of 10,922 patients with infectious diseases showed that the risk of sepsis was not associated with PCSK9 genetic variations [27]. Similarly, another study confirmed that there was no association between PCSK9 levels and resistance to antibiotics or the condition of patients in intensive care units [28]. In another human cohort, there was no correlation between plasma inflammation markers with total cholesterol levels, LDL cholesterol, and PCSK9 [28]. In a murine model, Berger et al. demonstrated that neither *PCSK9* KO nor PCSK9 antibodies protected mice against LPS-induced mortality [29]. Indeed, there are some disputes or even publication bias for these studies as they were based on different septic models. Meanwhile, there is still a lack of clinical trials focused on the potential mechanism. In the future, studies are required to investigate the potential mechanisms and links, in order to promote the application of PCSK9 in the treatment of sepsis.

## 4. Role of PCSK9 in the Pathogenesis of Sepsis

### 4.1. PCSK9 and LDL-R/VLDL-R

#### 4.1.1. Indirect Effects of PCSK9 on Inflammation

Pathogen lipids include the LPS from the wall of Gram-negative bacteria, lipoteichoic acid from the wall of the Gram-positive bacteria, and the mannan and phospholipid from the fungal wall. Pathogen lipids would be released into the peripheral circulation and bind with the transport proteins including lipopolysaccharide-binding protein (LBP), phospholipid transfer protein (PLTP), and cholesteryl ester transfer protein (CETP) [30]. The pathogen lipid-binding transport proteins could deliver the pathogen lipids to the innate immune cells and then trigger the inflammation through binding to Toll-like receptor (TLR) on the cellular surface of the innate immunocytes. Then, it would promote the release of various inflammatory mediators, which contributed to the defense and clearance of pathogens. Meanwhile, the pathogen lipid-binding transport protein could bind with the HDL particles, and then, the HDL-carried pathogen lipids were delivered to the LDL, VLDL, and chylomicron [31]. These particles were taken in by the LDL-R receptor on the surface of the hepatocytes or may be sequestered in adipose tissue by the VLDL-R. In this regard, PCSK9 may play a crucial role in amplifying the inflammation response by interfering with the clearance or isolation of pathogen lipids from the peripheral tissues [32]. In another study, PCSK9 could affect the pathogen lipid clearance and the secretion of downstream inflammatory factors through binding with LDL-R and the subsequent lysosome degradation in the sepsis and septic shock patients, which finally affected the prognosis of sepsis patients [21].

Recently, more attention has been paid to the effects of PCSK9 and VLDL-R on the pathogen lipids in the setting of sepsis. Previous studies have revealed that septic patients with obesity are paradoxically better than those without [33]. The exact mechanisms are still not well defined [34]. In a previous study, Shimada et al. indicated that the LPS could be absorbed by the adipose tissues upon 6 hrs after LPS injection in mice, depending on the VLDL-R rather than LDL-R. In the adipocyte cell lines, downregulation of VLDL-R mediated by PCSK9 protein leads to the decline of absorbance of LPS by the adipose cells. Moreover, there was significant improvement in the 90-day survival in the *VLDLR rs7852409* septic patients with at least one *LOF* allele of *PCSK9* compared with those without *PCKS9 LOF* alleles. This implied that the decline of PCSK9 may involve in the counterbalance of the damages induced by the allele mutation in VLDL-R [35].

### 4.2. PCSK9 and TLR4

#### 4.2.1. Direct Effect of PCSK9 on Inflammation

**Figure 1:**
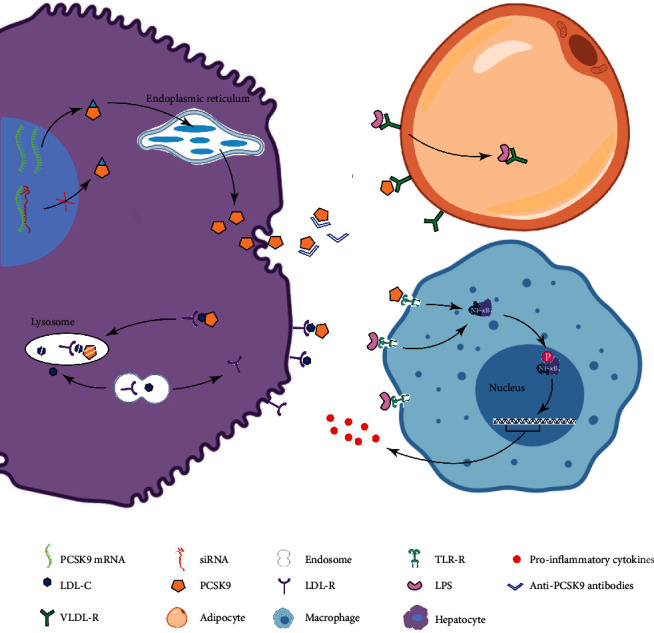
Roles of PCSK9 in the cholesterol metabolism and inhibitors in the hepatocytes, adipocyte, and macrophages. PCSK9 in the hepatocytes was catalyzed in the endoplasmic reticulum, followed by secretion to the peripheral parts. Finally, it led to escorting LDL-C and LDL-R to lysosome for degradation. The PCSK9 siRNA and the antibody could trigger the decline of PCSK9 amount in the intracellular and extracellular regions. VLDL-R sequesters lipopolysaccharide into adipose tissue during sepsis, while PCSK9 could downregulate the number of VLDL-R in the adipocytes, which then affected the uptake of LPS by the adipose tissues. The PCSK9 could activate the inflammation through the TLR4-activated NF-*κ*B signaling pathway. Some of the PCSK9 inhibitors were in clinical trials or on the market, including anti-PCSK9 antibody and siRNA.

As the crucial factor regulating the LDL-R activity, PCSK9 has been reported to involve in the cholesterol metabolism and then affect the clearance of pathogen lipids and the potential inflammation. Nevertheless, recent studies indicated that PCSK9 could directly mediate inflammatory response, independent of the cholesterol metabolism signaling pathway in the liver. For example, in the HepG2 cellular model, PCSK9 could affect the inflammatory reactions and was not associated with the cholesterol metabolism [36]. Meanwhile, in a clinical trial, a high serum level of PCSK9 was associated with the formation of plaque and inflammation in the coronary artery, with no correlation with the serum LDL-C [37]. Tang et al. indicated that the overexpression of PCSK9 in the macrophages could enhance the secretion of cytokines induced by ox-LDL. This study further confirmed that PCSK9 could enhance the secretion of inflammatory factors through modulating the expression of TLR-4/NF-*κ*B [38]. The exact mechanism may be related to the fact that there was similarity between the C-terminus of PCSK9 of the cysteine-rich domain and resistin [39]. Resistin could upregulate the expression of TLR-4 through binding to the TLR-4 by the C-terminus, which finally activated the TLR-4 signaling pathway [40, 41]. As a member of TLR receptor family, TLR-4 could recognize the LPS produced by the Gram-negative bacteria. In the setting of Gram-negative bacterial infection, the TLR-4-mediated LPS-TLR4-NF-*κ*B signaling pathway plays an important role in inducing the release of inflammatory mediators such as TNF-*α* and IL-6 [42] to involve in the anti-infection and inflammatory reactions by the innate immunocytes (e.g., mononuclear macrophages). In animal models, LPS could regulate the expression of PCSK9 mRNA [43]. Interestingly, in a recent study, significant elevation was found in PCSK9 and inflammatory factors in the rat model of alcohol liver disease, whereas there was a significant inhibition of inflammatory reaction after administration of anti-PCSK9 agents [44]. Thereby, it was proposed that PCSK9 may directly trigger the inflammatory reactions in the pathogenesis of alcohol liver disease. Nevertheless, further studies are required to investigate the potential mechanisms (Figure 1).

Moreover, unlike the signaling pathways mediated by LDL-R, PCSK9 was upregulated under inflammatory environments. For example, in the presence of Gram-negative bacterial infection, LPS could obviously upregulate the expression of PCSK9 [43], while PCSK9 could further enhance the inflammatory reactions through modulating the TLR-4/NF-*κ*B signaling pathway [38]. Severe SIRS is an important sign for the pathogenic bacterial-related infection (e.g., sepsis), which may trigger the tissue injury in vivo and the subsequent poor prognosis (Table 1).

## 5. PCSK9 Inhibitor

Currently, the anti-PCSK9 agents under development are mainly targeted at the blood lipid metabolism. There are mainly four types of PCSK9 inhibitors, including the monoclonal antibodies, small interfering RNA (siRNA), antisense oligonucleotides, and PCSK9 vaccines. The monoclonal antibodies, evolocumab, alirocumab, and bococizumab, can bind with PCSK9 and then block the interaction with LDL-R and neutralize the activity of PCSK9. It is noteworthy that evolocumab and alirocumab are humanized antibodies and superior to the other nonhumanized antibodies (e.g., bococizumab) as to the efficiency and safety. To date, two monoclonal antibodies (alirocumab, Praluent®; Sanofi/Regeneron; evolocumab, Repatha®, Amgen) have been approved by the FDA, which can safely reduce the LDL-C level [45–47]. Meanwhile, the majority of patients that received administration of bococizumab showed a high antiagent antibody titer, which then significantly offset the therapeutic effects of the agents. Even for those with no antidrug antibody formation, there was significant variation in the therapeutic effects of bococizumab. On this basis, the trial was terminated in 2016 [48]. The siRNA, a type of single-stranded RNA with a short length, could directly degrade the mRNA sequences that were targeted specifically by PCK9, which then inhibited the generation of PCSK9 [49]. In a phase I trial, PCSK9 synthesis inhibition induced by RNA interference (RNAi) was reported to provide a potentially safe mechanism to reduce LDL cholesterol concentration in healthy individuals with high level of cholesterols [50]. To date, PCSK9 siRNA (Inclisiran, Medicines) had entered the phase III clinical trial and showed excellent treatment efficiency [51]. In regard to antisense oligonucleotides, they could specifically bind with the PCSK9 target gene to inhibit the transcription of mRNA, which then resulted in reduction of PCSK9 secretion and the subsequent elevation of hepatic LDL-R as well as decline of LDL-C. However, there were still some disadvantages for the application of antisense oligonucleotides, especially the *in vivo* stability, which may affect the efficacy. The PCSK9 vaccine could induce the generation of anti-PCSK9 antibody, which then resulted in persistent elevation of PCSK9 antibody *in vivo*. Meanwhile, it could obviously bring down the level of LDL-C [52]. Particularly, the anti-PCSK9 agents under development are mainly focused on the blood lipid metabolism; however, no studies have been conducted to investigate the treatment efficiency of these agents in the setting of bacterial infection and sepsis. It requires further studies on this field.

## 6. Conclusion

Despite the fact that there are some disputes on the efficiency of PCSK9, massive studies indicated that PCSK9 could regulate the quantity of LDL-R/VLDL-R to affect the clearance or isolation of pathogen lipids mediated by LDL-R/VLDL-R. This would affect the prognosis of patients with bacterial infection. Some clinical studies demonstrated an association between the disease prognosis and serum PCSK9 as well as the presence of *PSCK9 LOF* allele. Meanwhile, inhibition of the PCSK9 production or function may improve the prognosis in animal model experiments. Therefore, PCSK9 may be a potential target for the treatment of sepsis.

Unfortunately, the exact mechanisms are still not clear. After taking the benefits of PCSK9 inhibitor in the blood lipid metabolism into consideration, great strides have been made in the research and development of PCSK9 inhibitors. However, there are no studies focused on the treatment of sepsis using PCSK9 in clinical settings. To date, the multiple clinical trials on the statins indicated that there were still disputes on the treatment efficiency [53]. We speculated that it may be related to the upregulation of PCSK9 mediated by hepatic LDL-R expression. Therefore, PCSK9 inhibitors were considered promising candidates for the pathogenic bacterial infection. PCSK9 could regulate the pathogen lipid clearance and inflammatory reactions by modulating the LDL-R signaling pathway, which served as a critical regulator of the innate immune response and septic shock outcome [21]. In addition, it could enhance the inflammatory reactions through modulating the TLR-4/NF-*κ*B signaling pathways in the inherent immunocytes, which was closely related to the atherosclerotic inflammation promotion [38].

Recently, inflammatory environment was reported to induce the generation of PCSK9 in the macrophages. In addition, the presence of PCSK9 would directly trigger the inflammation mediated by monocytes and the macrophages [54, 55]. Thus, we speculated that agents with the capacity of external and internal inhibition of PCSK9 (e.g., siRNA agents) may be more efficient to the external inhibition of PCSK9 (e.g., monoclonal antibody) in controlling the inflammation. In the future, these deserve more studies to further illustrate the potential mechanism.

## Figures and Tables

**Table 1:** A list of researches of effects of PCSK9 on sepsis.

Participants/model of study	Study type	Main results	Reference
Patients with sepsis	A single-centre observational cohort study	Plasma PCSK9 levels are greatly increased in sepsis. During sepsis, PCSK9 levels are highly correlated with the development of subsequent multiple organ failure.	Boyd et al. [20]
Male Pcsk9 knockout (Pcsk9^−/−^) mice Patients with septic shock (VASST derivation cohort/SPH validation cohort) Healthy, nonobese subjects GENE population	An experimental study Muticentre observational cohort studies	Reduced PCSK9 function is associated with increased pathogen lipid clearance via the LDL-R, a decreased inflammatory response, and improved septic shock outcome.	Walley et al. [21]
Patients with sepsis or sepsis shock	A retrospective observational study	Patients with multiple PCSK9 LOF alleles had lower risk of 1-year death or infection-related readmission compared with WT/single LOF groups.	Genga et al. [22]
Wild-type, PCSK9 knockout (KO), and PCSK9 transgenic mice	An experimental study	PCSK9 deficiency confers protection against systemic bacterial dissemination, organ pathology, and tissue inflammation, particularly in the lungs and liver, while PCSK9 overexpression exacerbates multiorgan pathology as well as the hypercoagulable and proinflammatory states in early sepsis.	Dwivedi et al. [25]
Patients with Gram-positive septic shock	A multicentre observational cohort study	Patients with PCSK9 LOF allele had significantly higher 28-day survival than those with no LOF alleles.	Leung et al. [26]
Patients hospitalized with infection	A retrospective cohort study	PCSK9 genetic variants were not significantly associated with risk of sepsis or the outcomes (cardiovascular failure and in-hospital death) of sepsis.	Feng et al. [27]
Patients with bacterial infections admitted to intensive care units	A cross-sectional study	There was no significant association between PCSK9 levels and either the severity of disease (APACHE II, SOFA, and GCS) indices or resistance to antibiotics.	Jamialahmadi et al. [28]
Female C57BL/6 mice	An experimental study	Inflammation state (LPS, zymosan, turpentine, etc.) stimulates PCSK9 expression	Feingold et al. [43]
C57Bl/6J wild-type and Ldlr-/- mice Pcsk9+/+ and Pcsk9-/- mice	An experimental study	PCSK9 inhibition provides no protection from LPS-induced mortality in mice.	Berger et al. [29]
Patients with septic shock	A subanalysis of the Albumin Italian Outcome Sepsis (ALBIOS) study	Patients with septic shock presenting with lower plasma PCSK9 levels experienced higher mortality rate.	Vecchié et al. [23]

The table summarized the experimental and clinical evidence on the role of PCSK9 and sepsis. PCSK9: proprotein convertase subtilisin/kexin type 9; LDL-R: low-density lipoprotein cholesterol receptors; KO: knockout; WT: wild type; LOF: loss of function; LPS: lipopolysaccharide.

## Data Availability

All the data are available upon appropriate request.

## References

[1] Singer M., Deutschman C. S., Seymour C. W. (2016). The third international consensus definitions for sepsis and septic shock (Sepsis-3). *JAMA*.

[2] Moore K. J., Tabas I. (2011). Macrophages in the pathogenesis of atherosclerosis. *Cell*.

[3] Stewart C. R., Stuart L. M., Wilkinson K. (2010). CD36 ligands promote sterile inflammation through assembly of a Toll-like receptor 4 and 6 heterodimer. *Nature Immunology*.

[4] Fessler M. B., Parks J. S. (2011). Intracellular lipid flux and membrane microdomains as organizing principles in inflammatory cell signaling. *The Journal of Immunology*.

[5] Benjannet S., Rhainds D., Essalmani R. (2004). NARC-1/PCSK9 and its natural Mutants. *Journal of Biological Chemistry*.

[6] Maxwell K. N., Breslow J. L. (2004). Adenoviral-mediated expression of Pcsk 9 in mice results in a low-density lipoprotein receptor knockout phenotype. *Proceedings of the National Academy of Sciences*.

[7] Seidah N. G., Benjannet S., Wickham L. (2003). The secretory proprotein convertase neural apoptosis-regulated convertase 1 (NARC-1): liver regeneration and neuronal differentiation. *Proceedings of the National Academy of Sciences of the United States of America*.

[8] Dixon D. L., Trankle C., Buckley L. (2016). A review of PCSK9 inhibition and its effects beyond LDL receptors. *Journal of Clinical Lipidology*.

[9] Ferdinand K. C., Nasser S. A. (2015). PCSK9 inhibition: discovery, current evidence, and potential effects on LDL-C and Lp(a). *Cardiovascular Drugs and Therapy*.

[10] Jeong H. J., Lee H. S., Kim K. S., Kim Y. K., Yoon D., Park S. W. (2008). Sterol-dependent regulation of proprotein convertase subtilisin/kexin type 9 expression by sterol-regulatory element binding protein-2. *Journal of Lipid Research*.

[11] Guo Y., Liu Q., Xu D. (2016). Shedding light on FGF21: a potential negative regulator of PCSK9. *International Journal of Cardiology*.

[12] Pitts R., Eckel R. (2014). The emerging role of PCSK9 inhibitors in preventive cardiology. *European Cardiology Review*.

[13] Brown M., Ahmed S. (2019). Emerging role of proprotein convertase subtilisin/kexin type-9 (PCSK-9) in inflammation and diseases. *Toxicology and Applied Pharmacology*.

[14] Humphries S. E., Whittall R. A., Hubbart C. S. (2006). Genetic causes of familial hypercholesterolaemia in patients in the UK: relation to plasma lipid levels and coronary heart disease risk. *Journal of Medical Genetics*.

[15] Cohen J. C., Boerwinkle E., Mosley T. H., Hobbs H. H. (2006). Sequence variations in PCSK9, low LDL, and protection against coronary heart disease. *The New England Journal of Medicine*.

[16] Dong B., Wu M., Li H. (2010). Strong induction of PCSK9 gene expression through HNF1alpha and SREBP2: mechanism for the resistance to LDL-cholesterol lowering effect of statins in dyslipidemic hamsters. *Journal of Lipid Research*.

[17] Francone O. L., Evangelista L., Fielding C. J. (1996). Effects of carboxy-terminal truncation on human lecithin:cholesterol acyltransferase activity. *Journal of Lipid Research*.

[18] Mayne J., Dewpura T., Raymond A. (2008). Plasma PCSK9 levels are significantly modified by statins and fibrates in humans. *Lipids in Health and Disease*.

[19] Careskey H. E., Davis R. A., Alborn W. E., Troutt J. S., Cao G., Konrad R. J. (2008). Atorvastatin increases human serum levels of proprotein convertase subtilisin/kexin type 9. *Journal of Lipid Research*.

[20] Boyd J. H., Fjell C. D., Russell J. A., Sirounis D., Cirstea M. S., Walley K. R. (2016). Increased plasma PCSK9 levels are associated with reduced endotoxin clearance and the development of acute organ failures during sepsis. *Journal of Innate Immunity*.

[21] Walley K. R., Thain K. R., Russell J. A. (2014). PCSK9 is a critical regulator of the innate immune response and septic shock outcome. *Science Translational Medicine*.

[22] Genga K. R., Lo C., Cirstea M. S. (2018). Impact of PCSK9 loss-of-function genotype on 1-year mortality and recurrent infection in sepsis survivors. *eBioMedicine*.

[23] Vecchié A., Bonaventura A., Meessen J. (2020). PCSK9 is associated with mortality in patients with septic shock: data from the ALBIOS study. *Journal of Internal Medicine*.

[24] Ruscica M., Ferri N., Fogacci F. (2017). Circulating levels of proprotein convertase subtilisin/kexin type 9 and arterial stiffness in a large population sample: data from the Brisighella Heart Study. *Journal of the American Heart Association*.

[25] Dwivedi D. J., Grin P. M., Khan M. (2016). Differential expression of PCSK9 modulates infection, inflammation, and coagulation in a murine model of sepsis. *Shock*.

[26] Leung A. K., Genga K. R., Topchiy E. (2019). Reduced proprotein convertase subtilisin/kexin 9 (PCSK9) function increases lipoteichoic acid clearance and improves outcomes in Gram positive septic shock patients. *Scientific reports*.

[27] Feng Q., Wei W. Q., Chaugai S. (2019). A Genetic approach to the association between PCSK9 and sepsis. *JAMA network open*.

[28] Jamialahmadi T., Panahi Y., Safarpour M. A. (2019). Association of serum PCSK9 levels with antibiotic resistance and severity of disease in patients with bacterial infections admitted to intensive care units. *Journal of clinical medicine*.

[29] Berger J. M., Loza Valdes A., Gromada J., Anderson N., Horton J. D. (2017). Inhibition of PCSK9 does not improve lipopolysaccharide-induced mortality in mice. *Journal of Lipid Research*.

[30] Yamashita S., Sakai N., Hirano K. I. (2001). Roles of plasma lipid transfer proteins in reverse cholesterol transport. *Frontiers in Bioscience*.

[31] Hailman E., Albers J. J., Wolfbauer G., Tu A. Y., Wright S. D. (1996). Neutralization and transfer of lipopolysaccharide by phospholipid transfer protein. *The Journal of Biological Chemistry*.

[32] Gautier T., Lagrost L. (2011). Plasma PLTP (phospholipid-transfer protein): an emerging role in 'reverse lipopolysaccharide transport' and innate immunity. *Biochemical Society Transactions*.

[33] Wacharasint P., Boyd J. H., Russell J. A., Walley K. R. (2013). One size does not fit all in severe infection: obesity alters outcome, susceptibility, treatment, and inflammatory response. *Critical care*.

[34] Ng P. Y., Eikermann M. (2017). The obesity conundrum in sepsis. *BMC Anesthesiology*.

[35] Shimada T., Topchiy E., Leung A. K. K. (2020). Very low density lipoprotein receptor sequesters lipopolysaccharide into adipose tissue during sepsis. *Critical Care Medicine*.

[36] Lan H., Pang L., Smith M. M. (2010). Proprotein convertase subtilisin/kexin type 9 (PCSK9) affects gene expression pathways beyond cholesterol metabolism in liver cells. *Journal of Cellular Physiology*.

[37] Cheng J. M., Oemrawsingh R. M., Garcia-Garcia H. M. (2016). PCSK9 in relation to coronary plaque inflammation: results of the ATHEROREMO-IVUS study. *Atherosclerosis*.

[38] Tang Z. H., Peng J., Ren Z. (2017). New role of PCSK9 in atherosclerotic inflammation promotion involving the TLR4/NF-*κ*B pathway. *Atherosclerosis*.

[39] Hampton E. N., Knuth M. W., Li J., Harris J. L., Lesley S. A., Spraggon G. (2007). The self-inhibited structure of full-length PCSK9 at 1.9 A reveals structural homology with resistin within the C-terminal domain. *Proceedings of the National Academy of Sciences of the United States of America*.

[40] Tarkowski A., Bjersing J., Shestakov A., Bokarewa M. I. (2010). Resistin competes with lipopolysaccharide for binding to toll-like receptor 4. *Journal of Cellular and Molecular Medicine*.

[41] Kang S. W., Kim M. S., Kim H. S. (2013). Celastrol attenuates adipokine resistin-associated matrix interaction and migration of vascular smooth muscle cells. *Journal of Cellular Biochemistry*.

[42] Xia Q., Wei L., Zhang Y., Sheng J., Wu W., Zhang Y. (2018). Immune checkpoint receptors Tim-3 and PD-1 regulate monocyte and T lymphocyte function in septic patients. *Mediators of Inflammation*.

[43] Feingold K. R., Moser A. H., Shigenaga J. K., Patzek S. M., Grunfeld C. (2008). Inflammation stimulates the expression of PCSK9. *Biochemical and Biophysical Research Communications*.

[44] Lee J. S., Mukhopadhyay P., Matyas C. (2019). PCSK9 inhibition as a novel therapeutic target for alcoholic liver disease. *Scientific reports*.

[45] McKenney J. M. (2015). Understanding PCSK9 and anti-PCSK9 therapies. *Journal of Clinical Lipidology*.

[46] Sabatine M. S., Giugliano R. P., Wiviott S. D. (2015). Efficacy and safety of evolocumab in reducing lipids and cardiovascular events. *The New England Journal of Medicine*.

[47] Robinson J. G., Farnier M., Krempf M. (2015). Efficacy and safety of alirocumab in reducing lipids and cardiovascular events. *The New England Journal of Medicine*.

[48] Ballantyne C. M., Neutel J., Cropp A. (2015). Results of bococizumab, a monoclonal antibody against proprotein convertase subtilisin/kexin type 9, from a randomized, placebo-controlled, dose-ranging study in statin-treated subjects with hypercholesterolemia. *The American Journal of Cardiology*.

[49] Lam J. K., Chow M. Y., Zhang Y., Leung S. W. (2015). siRNA versus miRNA as therapeutics for gene silencing. *Molecular Therapy-Nucleic Acids*.

[50] Fitzgerald K., Frank-Kamenetsky M., Shulga-Morskaya S. (2014). Effect of an RNA interference drug on the synthesis of proprotein convertase subtilisin/kexin type 9 (PCSK9) and the concentration of serum LDL cholesterol in healthy volunteers: a randomised, single-blind, placebo-controlled, phase 1 trial. *Lancet*.

[51] Iacobucci G. (2020). Inclisiran: UK to roll out new cholesterol lowering drug from next year. *BMJ*.

[52] Landlinger C., Pouwer M. G., Juno C. (2017). The AT04A vaccine against proprotein convertase subtilisin/kexin type 9 reduces total cholesterol, vascular inflammation, and atherosclerosis in APOE∗3Leiden.CETP mice. *European Heart Journal*.

[53] Thomas G., Hraiech S., Loundou A. (2015). Statin therapy in critically-ill patients with severe sepsis: a review and meta-analysis of randomized clinical trials. *Minerva Anestesiologica*.

[54] Tang Z., Jiang L., Peng J. (2012). PCSK9 siRNA suppresses the inflammatory response induced by oxLDL through inhibition of NF-*κ*B activation in THP-1-derived macrophages. *International Journal of Molecular Medicine*.

[55] Ricci C., Ruscica M., Camera M. (2018). PCSK9 induces a pro-inflammatory response in macrophages. *Scientific reports*.

